# Lockdown Measures Against the Spread of the COVID-19 Pandemic: Negative Effects for People Living With Depression

**DOI:** 10.3389/fpsyg.2022.789173

**Published:** 2022-02-04

**Authors:** Andreas Czaplicki, Hanna Reich, Ulrich Hegerl

**Affiliations:** ^1^German Depression Foundation, Leipzig, Germany; ^2^Depression Research Center of the German Depression Foundation, Department for Psychiatry, Psychosomatics and Psychotherapy, Goethe University, Frankfurt, Germany; ^3^Johann Christian Senckenberg Distinguished Professorship, Department for Psychiatry, Psychosomatics and Psychotherapy, Goethe University, Frankfurt, Germany

**Keywords:** COVID-19, healthcare, depression, depression-related lifestyle, loss of daily structure, lack of exercise, extended bed and sleep times

## Abstract

The COVID-19 pandemic and associated measures to restrict the spread of the virus correlated with limitations in healthcare and changes in depression-related lifestyle elements (loss of daily structure, lack of exercise, and extended bed and sleep time) for depressed patients, both of which are known to negatively affect the course of depression. This paper examines, (i) the reporting of a worsening state of illness as a result of COVID-19-related measures among individuals with depressive disorders; and (ii) whether this worsening was related to restrictions in healthcare for depression or changes in depression-related lifestyle. The analysis was based on a population-representative survey of the German population aged 18–69 years (*N* = 5,135 respondents, comprising a subgroup of *n* = 1,038 persons suffering from depression and *n* = 598 persons who spent the lockdown primarily in home isolation). The key findings were: 49% (*n* = 505) of respondents with self-reported diagnosed depression reported that the measures against the pandemic had a negative impact on their depressive illness (new depressive episode, worsening of symptoms, suicidal impulses, suicide attempt, and other negative consequences). Of those who reported impaired access to healthcare for their depressive illness, 70% (*n* = 276) also reported a worsening of their depressive illness. This was a significantly higher percentage than those who did not experience impaired access to healthcare (36%, *n* = 229, *p* < 0.001). Of those who reported changes in depression-related lifestyle (loss of daily structure, lack of exercise, or extended bed and sleep time), 58% (*n* = 308) reported a worsening of their depressive illness. This was a significantly higher percentage than those who did not exhibit any of the outlined behaviours (28%, *n* = 19, *p* < 0.001). Worsening of the depressive illness was most common among those who reported a lack of daily structure or extended bedtimes (67%; *n* = 230 resp. *N* = 226). People who mentioned a lack of exercise also reported a worsening of their depressive illness (59%; *n* = 271). These findings reinforce the need to consider the suffering and possible increased suicide risk to people suffering from depression induced by measures designed to constrain the COVID-19 pandemic; an important consideration in identifying the optimal risk-benefit ratio when setting pandemic measures. Our study highlighted the importance of maintaining healthcare, even in crisis situations, and ensuring access to guideline-based treatment for people who need urgent care. It also showed that political interventions can influence individual behaviours that can have negative effects on depressive illness.

## Introduction

To contain the COVID-19 pandemic in Germany, in March 2020 widespread restrictions on public life were put in place across the country and remained in place until May/June of 2021. These included lockdown, distancing and hygiene rules, mandatory use of masks, curfew restrictions, closure of shops, home office for many employees, home schooling for students, and more (Bundesanzeiger, 2021; Deutscher Bundestag, 2021).

Broad effects of the pandemic on mental wellbeing have been documented (Ettman et al., 2020; González-Sanguino et al., 2020; Gualano et al., 2020; Pierce et al., 2020; Sønderskov et al., 2020). However, most of these can be considered as healthy or non-clinical reactions to stressors associated with the pandemic and with the measures taken against it. Non-clinical depressive symptoms need to be distinguished from depressive disorders. Recent studies showed that depressive disorders increased during the pandemic. The authors of a meta-analysis estimate that there are 53.2 million additional cases of major depressive disorder worldwide due to the COVID-19 pandemic; this represents a 28% increase (Santomauro et al., 2021). In Germany a survey of 15,037 people aged 18 years and older showed a 14.3% increase in depressive symptoms (Bäuerle et al., 2020). A retrospective cross-sectional study based on health insurance data showed a 12% increase in diagnoses of depressive illness in children and adolescents aged 2–17 years for the period April to December 2002 compared to the previous year (Kostev et al., 2021). A member survey conducted by the German Psychotherapists Association in January 2021 showed that the number of inquiries in psychotherapeutic practices had increased by about 40%, when compared to the previous year (Rabe-Menssen, 2021). In Germany, there has been a long-term downward trend in suicides. However in 2020, a rise to 9,206 suicides, up from 9,041 suicides in 2019, occurred (Gesundheitsberichterstattung des Bundes, 2021).

The present analyses focus on individuals suffering from depression, in this vulnerable subgroup the negative effects of pandemic associated stressors are for the following reasons, to be expected:

i) The medical care for patients with depressive disorders has deteriorated, as resources of the healthcare system have been kept free for patients with COVID-19, outpatient clinics have reduced their services, self-help groups were cancelled, and depressed patients have cancelled appointments with doctors and psychotherapists on their own accord because of fears of infection. In Germany, 35% of the general population surveyed stated that they had been affected by restrictions in their medical care (either their care or that of a close relative) during the first lockdown. Among survey participants in particular need of treatment due to a current depressive phase of illness, the reported limitations were 56%. In June/July 2020, this figure was 22% of all respondents in the general population, or 42% of people with a current depressive phase of illness (Reich et al., 2021).
ii) There are changes in depression-related lifestyle which are known to have negative effects on the course of depression. Among these are the loss of daily structure (Peters et al., 2021), lack of exercise (Stathopoulou et al., 2006; Krogh et al., 2011; Helgadóttir et al., 2015), and extended bed and sleep times (Lorenz et al., 2020).

The impact of the lockdown on depressive illness, on healthcare for depression and on changes in depression-related lifestyle, have so far been considered in isolation and, as far as we know, not analysed in the context of the COVID-19 pandemic. Therefore, this article examines the following questions:

1. Do people with a depressive disease report a worsening of their disease state due to the measures implemented to contain the COVID-19 pandemic?
2. Is patient reported worsening of depressive disorders related to restrictions in healthcare for depression, due to the measures implemented to contain the COVID-19 pandemic?
3. Is patient reported worsening of depressive disorders related to changes in depression-related lifestyle (loss of daily structure, lack of exercise, and extended bed and sleep times) during the lockdown and if this is the case, which aspect is most important?

## Materials and Methods

The analysis was based on a population-representative survey of the German resident population aged 18–69 years. The survey was conducted online by the certified survey company (ISO 26362) Respondi AG, the survey period was from 17 to 28 February 2021. The survey was conducted in a period when restrictions on public life were placed on the German population in the form of a second lockdown (starting December 2020). The population was called upon to minimise the risk of infection and contain the spread of the virus by adhering to specific rules (distance, hygiene, and daily masks).

The survey cohort was a multiple stratified quota selection based on the interleaved characteristics of gender, age, federal state/group of federal states according to the current population updates of the Federal Statistical Office of Germany. Respondents were recruited from a pool of people who declared their general willingness to participate in anonymous surveys. These respondents had either been recruited offline or had registered in the online pool with their own initiative (self-recruited). Based on the specifications of a quota plan, participants of the different population segments were randomly contacted by electronic mail and asked to participate in the survey. This was done in several stages. If the target values of the quota cells were reached, they were closed to further respondents. There is no evidence of self-selection on the topic of depression. The invitations to respondents were made without reference to the survey topic. After the topic of depression was mentioned, there was no increased dropout rate. The online sample forms a reduced image of the basic population and is thus representative of the German population aged 18–69 years. Due to minor deviations of the quota characteristics from the specifications, the data were weighted by adjusting the structure of the sample with regard to gender, age, federal state, to align to the data of the Federal Statistical Office of Germany. Each case was given an individual weight, which varied between 0.9655 and 1.0188 across all respondents. The sample thus corresponds to the composition of the total population.

### Survey Instruments

The identification of those suffering from depression was assessed *via* self-description (“Have you already come into contact with the disease depression?”). If participants chose the response option “Yes, I have already been diagnosed with depression once,” they were included in the analyses.

The worsening of the reported disease state associated with the measures to contain the spread of COVID-19 was asked as follows: “Have you experienced a deterioration in your health with regard to your depressive illness in the past 6 months as a result of the measures against Corona?” The possible answers were: “my depression has worsened,” “I had a relapse into a depressive episode,” “I had suicidal thoughts or impulses,” “I attempted suicide,” “there were other factors that worsened, with regard to my depressive illness.” As an exclusion of the above items, there was the option: “no, there was no worsening of my depression illness.” In the analysis, we only looked at people who reported a worsening of their disease.

The restrictions in medical care for depression were examined with the following question: “Did the situation due to the Corona crisis have an impact on the healthcare (treatments, therapies, support services) of your depression in the past 6 months?” Response options were: “my support group could no longer meet,” “my support group met virtually,” “treatment appointments with the specialist were cancelled,” “treatment appointments with the GP were cancelled,” “treatment appointments with the psychotherapist were cancelled,” “I could not get treatment appointments, although this would have helped me,” “I did not keep my treatment appointments because the situation was too uncertain for me,” “a planned inpatient stay could not take place,” “there were other restrictions in the care of my depression illness,” “no, there were no concrete effects.” In the analysis, we only looked at people who reported impairments in the medical deterioration (worsening) of their disease.

The changes in depression-related lifestyle were asked for a subgroup (598 out of 1,038 respondents) with self-reported depressive disorder in home isolation, using the following question: “You said that in the past 6 months you spent most of the day in the home environment and only left it in urgent cases. What effects have you observed in yourself?” Changes in the structure of the day were queried as follows: “My day no longer had any real structure; the whole routine had become disorganised.” Lack of exercise was queried with the statement: “I moved too little.” Two statements were used to determine the extended bedtime: “The time I spent in bed increased” and “I lay in bed more often during the day.” Agreement with these items was determined on a four-point scale: “strongly agree,” “agree,” “somewhat agree,” “disagree.”

### Sample

The survey comprised *N* = 5,135 people. Among them *n* = 1,038 (20.2%) self-reported to have been diagnosed with depression (*n* = 622 women, 59.9%). 11.8% (*n* = 122) of the respondents were in the 18–29 age group, 15.8% (*n* = 164) in the 30–39 age group, 20.6% (*n* = 214) in the 40–49 age group, 30.6% (*n* = 318) in the 50–59 age group and 21.2% (*n* = 220) in the 60–69 age group.

### Statistical Analysis

First, the worsening of the disease status, the restriction in healthcare for depressive disorders and the changes of depression-relevant lifestyle were considered in summary (occurred/did not occur). For the more in-depth analysis, the number of care restrictions and depression-relevant behaviours were examined. The two statements identifying an extended bedtime were analysed together. Chi-square tests were performed as a measure of the association (95% confidence interval), and Fisher’s Exact Test was calculated for tables with cell sizes smaller than 5. The data were analysed using SPSS (alpha = 0.05).

## Results

### Worsening of the Reported Disease State

49% (*n* = 505) of respondents with diagnosed depression reported that they experienced a worsening of their disease state as a consequence of the measures to restrict the spread of COVID-19. Of these, 18% (*n* = 186) had suffered from a new depressive episode, 20% (*n* = 209) reported a worsening of symptoms, 9% (*n* = 96) reported suicidal impulses, and individual respondents reported a suicide attempt (*n* = 4) within the preceding 6 months. In addition, 20% (*n* = 212) mentioned other negative consequences of the COVID-19 measures on their illness, which were not reported in more detail (see Table 1).

**TABLE 1:** Associations of COVID-19 restrictions and healthcare for depressive disorders.

		Restrictions in healthcare for depression			Number of restrictions in healthcare for depression				
	All depressive patients (*N* = 1,038)	Not restricted (*n* = 644)	Restricted (*n* = 394)		One (*n* = 181)	Two (*n* = 104)	Three (*n* = 53)	Four and more (*n* = 56)	
Negative effects on depressive illness (at least one of the following items named)	49% (*n* = 505)	**36%** **(*n* = 229)**	**70%** **(*n* = 276)**	*p* < 0.001	**65%** **(*n* = 117)**	**63%** **(*n* = 65)**	**83%** **(*n* = 44)**	**89%** **(*n* = 50)**	*p* < 001
“I had a relapse into a depressive episode”	18% (*n* = 186)	**13%** **(*n* = 86)**	**25%** **(*n* = 100)**	*p* < 0.001	**19%** **(*n* = 35)**	**17%** **(*n* = 18)**	**40%** **(*n* = 21)**	**46%** **(*n* = 26)**	*p* < 0.001
“My depression has gotten worse (worsening of depressive symptoms)”	20% (*n* = 209)	**10%** **(*n* = 62)**	**37%** **(*n* = 147)**	*p* < 0.001	**31%** **(*n* = 56)**	**29%** **(*n* = 30)**	**55%** **(*n* = 29)**	**57%** **(*n* = 32)**	*p* < 0.001
“I had suicidal thoughts or impulses”	9% (*n* = 96)	**5%** **(*n* = 35)**	**16%** **(*n* = 61)**	*p* < 0.001	**13%** **(*n* = 23)**	**11%** **(*n* = 11)**	**13%** **(*n* = 7)**	**36%** (***n* = 20)**	*p* < 0.001
“I have attempted suicide”	<1% (*n* = 4)	0% (*n* = 0)	1% (*n* = 4)	0.056	1% (*n* = 1)	0% (*n* = 0)	0% (*n* = 0)	5% (*n* = 3)	0.006
“There have been other deteriorations regarding my depressive illness”	20% (*n* = 212)	**15%** **(*n* = 97)**	**29%** **(*n* = 115)**	*p* < 0.001	**25%** **(*n* = 46)**	**25%** **(*n* = 26)**	**30%** **(*n* = 16)**	**48%** **(*n* = 27)**	*p* < 0.001

*Chi-square test, 95% significance level, for 2 × 2 tables with cell sizes <5 Fisher’s Exact Text was calculated, significant differences are highlighted in bold.*

### Relationship of Worsening Depressive Illness and Restrictions in Healthcare for Depression

Among respondents with a diagnosed depressive disorder that reported healthcare for their depressive disorder was restricted during the COVID-19 pandemic, a total of 70% (*n* = 276) reported worsening of their disease state. They report worsening of depressive symptoms (37%; *n* = 143), a recurrence into a depressive episode (25%; *n* = 100) and suicidal impulses (16%; *n* = 61). All four depressed patients who reported a suicide attempt indicated that healthcare for their depression had been impaired. In addition, other unspecified worsening of the depressive illness was reported by 29% (*n* = 115).

Among those who reported no restriction of healthcare for depression, only 36% (*n* = 229) reported a worsening of their disease state (*p* < 0.001). Only 10% (*n* = 62) reported a worsening of symptoms, only 13% (*n* = 86) a recurrence into a depressive episode, and only 5% (*n* = 36) suicidal impulses. None of them reported a suicide attempt. Other unspecified worsening of the depressive illness was reported by 15% of those whose care was not impaired (*n* = 97) (see Table 1).

### Relationship of Worsening Depressive Disorders and Changes in Depressive-Related Lifestyle

Among the 1,038 respondents with a self-reported depressive illness, 598 said they had largely stayed at home during the lockdown and hardly left the house. Among them, 58% (*N* = 308) of respondents who reported changes in depressive-related lifestyle (loss of daily structure, lack of exercise, or extended bed and sleep times) also mentioned negative effects on their depressive illness. Specifically, they experienced a relapse into a depressive episode significantly more often than respondents without changes in depression-related lifestyle (21%, *n* = 113 to 6%, *n* = 4). They were more likely to report a worsening of depressive symptoms (27%, *n* = 141 at 9%, *n* = 6), more likely to have suicidal impulses and thoughts (12%, *n* = 61 at 3%, *n* = 2), and more likely to report other worsening of their depressive illness (24%, *n* = 128 at 13%, *n* = 9). All three depressed patients who mainly stayed at home in the lockdown and reported a suicide attempt, indicated that their depression-related lifestyle in the lockdown was unfavourable for the illness. Among those with depression who did not exhibit any of the assessed behaviours in the lockdown, only 28% (*n* = 19) reported worsening of their depressive illness (*p* < 0.001).

The more extensive the change in depression-relevant lifestyle was in the lockdown, the more often respondents reported negative effects on the depressive illness. Among those who mentioned all three behaviours, 75% (*n* = 161) reported a worsening of their depression. If two of the three behavioural changes occurred, 55% (*n* = 97) reported a worsening of their depression. Among those who mentioned one of the behavioural changes, 36% (*n* = 50) reported worsening of their depressive illness (*p* < 0.001).

Worsening of the depressive disorder was most common among those who reported lack of daily structure or extended bedtimes (67%; *n* = 230 resp. *n* = 226). People who mentioned lack of exercise reported a worsening of their depressive illness by 59% (*n* = 271) (see Table 2).

**TABLE 2:** Associations of problematic depression-related lifestyle changes with depressive illness.

		Problematic depression-related lifestyle			Number of problematic depression-related lifestyle				Type of problematic depression-related lifestyle		
	All depressive patients (diagnosed) in home isolation (*n* = 598)	No (*n* = 67)	Yes (*n* = 531)		One (*n* = 140)	Two (*n* = 175)	Three (*n* = 216)		Loss of daily structure (*n* = 343)	Lack of exercise (*n* = 459)	Extended bed and sleep times (*n* = 336)
Negative effects on depressive illness (at least one of the following items named)	55% (*n* = 327)	**28%** **(*n* = 19)**	**58%** **(*n* = 308)**	*p* < 0.001	**36%** **(*n* = 50)**	**55%** **(*n* = 97)**	**75%** **(*n* = 161)**	*p* < 0.001	67% (*n* = 230)	59% (*n* = 271)	67% (*n* = 226)
“I had a relapse into a depressive episode”	20% (*n* = 117)	**6%** **(*n* = 4)**	**21%** **(*n* = 113)**	*p* < 0.001	**10%** **(*n* = 14)**	**20%** **(*n* = 35)**	**30%** **(*n* = 64)**	*p* < 0.001	24% (*n* = 83)	22% (*n* = 100)	28% (*n* = 93)
“My depression has gotten worse (worsening of depressive symptoms)”	25% (*n* = 147)	**9%** **(*n* = 6)**	**27%** **(*n* = 141)**	*p* < 0.001	**13%** **(*n* = 18)**	**21%** **(*n* = 37)**	**40%** **(*n* = 86)**	*p* < 0.001	33% (*n* = 114)	28% (*n* = 129)	32% (*n* = 107)
“I had suicidal thoughts or impulses”	11% (*n* = 63)	**3%** **(*n* = 2)**	**12%** **(*n* = 61)**	*p* < 0.018	**4%** **(*n* = 5)**	**11%** **(*n* = 20)**	**17%** **(*n* = 36)**	*p* < 0.001	15% (*n* = 52)	12% (*n* = 54)	14% (*n* = 47)
“I have attempted suicide”	1% (*n* = 3)	0% (*n* = 0)	1% (*n* = 3)	*p* < 0.700	0% (*n* = 0)	0% (*n* = 0)	1% (*n* = 3)	0.149	1% (*n* = 3)	1% (*n* = 3)	1% (*n* = 3)
“There have been other deteriorations regarding my depressive illness”	23% (*n* = 137)	**13%** **(*n* = 9)**	**24%** **(*n* = 128)**	0.05	**14%** **(*n* = 20)**	**21%** **(*n* = 37)**	**33%** **(*n* = 71)**	*p* < 0.001	29% (*n* = 99)	25% (*n* = 113)	28% (*n* = 95)

*Chi-square test, 95% significance level, for 2 × 2 tables with cell sizes <5 Fisher’s Exact Test was calculated, significant differences are highlighted in bold.*

## Discussion

The study shows that 49% of the survey respondents with depression reported a worsening of their illness due to the measures imposed during the COVID-19 pandemic. Clear associations were found between this worsening and participants’ reported impairments of healthcare as well as behavioural changes with known negative effects on depression. Of those who reported impaired medical care for their depressive illness, 70% (*n* = 276) reported a worsening of their depressive illness. Of those who reported changes in depression-related lifestyle (loss of daily structure, lack of exercise, or extended bed and sleep times), 58% (*n* = 308) reported a worsening of their depressive illness.

In part, health systems in Germany have reacted quickly to the COVID-19 pandemic, and as early as spring 2020, lifted the limit on video treatments that had been in place (prior to the pandemic) and expanded options for telephone consultations. As many as 15% of those suffering from depression stated that during the pandemic they had conducted consultations with a doctor or psychological psychotherapist by telephone or video for the first time. Patients also increasingly used digital care services. This can be seen in the user numbers of the digital iFightDepression (iFD) tool (Oehler et al., 2020). iFD had around 3,500 registered users accompanied by a guide (health professional) at the time of the first lockdown in March 2020. After the imposition of the lockdown and an anticipated negative impact of the associated consequences for people with depression, the tool was briefly made available for users without a guide. This meant that those affected could register directly and use the tool without the guidance of their health professional. Within a short time, around 14,000 people registered for and used the tool (Oehler et al., 2021).

It is not unlikely that these associations are the result of negative effects of the measures against the spread of COVID-19 on people with depressive disorders. However, the findings from this cross-sectional study does not allow conclusions concerning the causality behind the observed effects. Although the people did state that the behavioural changes presented were consequences of the measures, the possibility of an inverse causality cannot be ruled out. For example, those who experience worsening of their illness might have been more likely to seek treatment and were therefore also more likely to be affected by the limitations. Similarly, longer bedtimes, less exercise, and difficulties in structuring the day might have been caused by an increase in depression severity. While the findings describe the specific effects of the 2020/2021 lockdown, they should also prompt us to look ahead. The COVID-19 pandemic has shown how quickly such a crisis can lead to an impairment in the care of depressive disorders. We should for the future, develop structures and measures to maintain healthcare in comparable situations, to ensure access to guideline-compliant treatment for people in urgent need of treatment. Digital support services, self-help (Blume et al., 2009) and self-management applications (Oehler et al., 2020) can help to facilitate access to treatment and community when face-to-face contact needs to reduced. It may be even more difficult to mitigate the negative side effects of lockdown and domestic isolation. Initiation and upscaling of additional services, e.g., outdoor exercise therapy (Knapen et al., 2015), targeted at the most vulnerable groups may help to ensure that depressive patients in pandemic or public crisis situations do not drift into behaviour inducing a risk for an unfavourable course of their illness.

## Data Availability Statement

The raw data supporting the conclusions of this article will be made available by the authors, without undue reservation.

## Ethics Statement

Ethical review and approval was not required for the study on human participants in accordance with the local legislation and institutional requirements. Written informed consent for participation was not required for this study in accordance with the national legislation and the institutional requirements.

## Author Contributions

All authors listed have made a substantial, direct, and intellectual contribution to the work, and approved it for publication.

## Conflict of Interest

The authors declare that the research was conducted in the absence of any commercial or financial relationships that could be construed as a potential conflict of interest.

## Publisher’s Note

All claims expressed in this article are solely those of the authors and do not necessarily represent those of their affiliated organizations, or those of the publisher, the editors and the reviewers. Any product that may be evaluated in this article, or claim that may be made by its manufacturer, is not guaranteed or endorsed by the publisher.

## References

[B1] Bäuerle A. Teufel M. Musche V. Weismüller B. Kohler H. Hetkamp M. (2020). Increased generalized anxiety, depression and distress during the COVID-19 pandemic: a cross-sectional study in Germany. *J. Public Health* 42 672–678. 10.1093/pubmed/fdaa106 32657323 PMC7454766

[B2] Blume A. Mergl R. Niedermeier N. Kunz J. Pfeiffer-Gerschel T. Karch S. (2009). Evaluation of an online discussion forum for depressive patients and their relatives–an examination focussing motives and effects of participation. *Neuropsychiatrie* 23 42–51. 19272291

[B3] Bundesanzeiger (2021). Gesetz zur Fortgeltung der die Epidemische Lage von Nationaler Tragweite Betreffenden Regelungen. Vom 29. Bundesgesetzblatt Jahrgang 2021 Teil I Nr. 12, Ausgegeben zu Bonn am 30. März 2021, S. 370. Available online at: https://www.bgbl.de/xaver/bgbl/text.xav?SID=&tf=xaver.component.Text_0&tocf=&qmf=&hlf=xaver.component.Hitlist_0&bk=bgbl&start=%2F%2F*%5B%40node_id%3D%27910028%27%5D&skin=pdf&tlevel=-2&nohist=1 (accessed July 07, 2021).

[B4] Deutscher Bundestag (2021). *Entwurf eines Gesetzes zum Schutz der Bevölkerung bei einer Epidemischen Lage von Nationaler Tragweite, Gesetzentwurf der Fraktionen der CDU/CSU und SPD, Deutscher Bundestag, 19. Wahlperiode, Drucksache 19/18111 24.03.2020.* Available online at: https://dserver.bundestag.de/btd/19/181/1918111.pdf (accessed July 07, 2021).

[B5] Ettman C. K. Abdalla S. M. Cohen G. H. Sampson L. Vivier P. M. Galea S. (2020). Prevalence of depression symptoms in US adults before and during the COVID-19 pandemic. *JAMA Netw. Open* 3:e2019686. 10.1001/jamanetworkopen.2020.19686 32876685 PMC7489837

[B6] Gesundheitsberichterstattung des Bundes (2021). *Sterbefälle. Gliederungsmerkmale: Jahre, Region, Alter, Geschlecht, Familienstand, ICD-10.* Available online at: https://www.gbe-bund.de/gbe/pkg_isgbe5.prc_menu_olap?p_uid=gast&p_aid=47339234&p_sprache=D&p_help=3&p_indnr=670&p_indsp=&p_ityp=H&p_fid= (accessed November 23, 2021).

[B7] González-Sanguino C. Ausín B. Castellanos M. Á Saiz J. López-Gómez A. Ugidos C. (2020). Mental health consequences during the initial stage of the 2020 coronavirus pandemic (COVID-19) in Spain. *Brain Behav. Immun.* 87 172–176. 10.1016/j.bbi.2020.05.040 32405150 PMC7219372

[B8] Gualano M. R. Lo Moro G. Voglino G. Bert F. Siliquini R. (2020). Effects of Covid-19 lockdown on mental health and sleep disturbances in Italy. *Int. J. Environ. Res. Public Health* 17:4779. 10.3390/ijerph17134779 32630821 PMC7369943

[B9] Helgadóttir B. Forsell Y. Ekblom Ö (2015). Physical activity patterns of people affected by depressive and anxiety disorders as measured by accelerometers: a cross-sectional study. *PLoS One* 10:e0115894. 10.1371/journal.pone.0115894 25585123 PMC4293141

[B10] Knapen J. Vancampfort D. Moriën Y. Marchal Y. (2015). Exercise therapy improves both mental and physical health in patients with major depression. *Disabil. Rehabil.* 37 1490–1495. 25342564 10.3109/09638288.2014.972579

[B11] Kostev K. Weber K. Riedel-Heller S. von Vultée C. Bohlken J. (2021). Increase in depression and anxiety disorder diagnoses during the COVID-19 pandemic in children and adolescents followed in pediatric practices in Germany. *Eur. Child Adolesc. Psychiatry* 1–7. 10.1007/s00787-021-01924-1 34825964 PMC8619647

[B12] Krogh J. Nordentoft M. Sterne J. A. Lawlor D. A. (2011). The effect of exercise in clinically depressed adults: systematic review and meta-analysis of randomized controlled trials. *J. Clin. Psychiatry* 72 529–538. 21034688 10.4088/JCP.08r04913blu

[B13] Lorenz N. Sander C. Ivanova G. Hegerl U. (2020). Temporal associations of daily changes in sleep and depression core symptoms in patients suffering from major depressive disorder: idiographic time-series analysis. *JMIR Ment. Health* 7:e17071. 10.2196/17071 32324147 PMC7206522

[B14] Oehler C. Görges F. Rogalla M. Rummel-Kluge C. Hegerl U. (2020). Efficacy of a guided web-based self-management-intervention for depression or dysthymia: randomized controlled trial with a 12-month follow-up using an active control condition. *J. Med. Internet Res.* 22:e15361. 10.2196/15361 32673233 PMC7388040

[B15] Oehler C. Scholze K. Reich H. Sander C. Hegerl U. (2021). Intervention use and symptom change with unguided internet-based cognitive behavioral therapy for depression during the COVID-19 pandemic: log data analysis of a convenience sample. *JMIR Ment. Health* 8:e28321. 34115604 10.2196/28321 PMC8288646

[B16] Peters E. Hübner J. Katalinic A. (2021). Stress Copingstrategien und gesundheitsbezogene Lebensqualität während der Corona-Pandemie im April 2020 in Deutschland [Stress, coping strategies and health-related quality of life during the corona pandemic in April 2020 in Germany]. *Dtsch. Med. Wochenschr.* 146 e11–e20. 10.1055/a-1275-3792 33260231 PMC7815330

[B17] Pierce M. Hope H. Ford T. Hatch S. Hotopf M. John A. (2020). Mental health before and during the COVID-19 pandemic: a longitudinal probability sample survey of the UK population. *Lancet Psychiatry* 7 883–892. 10.1016/S2215-0366(20)30308-4 32707037 PMC7373389

[B18] Rabe-Menssen C. (2021). *Patientenanfragen während der Corona-Pandemie.* Berlin: DPtV, Deutsche PsychotherapeutenVereinigung.

[B19] Reich H. Czaplicki A. Gravert C. Hegerl U. (2021). Negative Effekte der COVID-19-Maßnahmen auf die Versorgung depressiv Erkrankter. Ergebnisse einer repräsentativen Bevölkerungsbefragung. *Nervenarzt.* 10.1007/s00115-021-01148-3 34137902 PMC8210502

[B20] Santomauro D. F. Mantilla Herrera A. M. Shadid J. Zheng P. Ashbaugh C. Pigott D. M. (2021). Global prevalence and burden of depressive and anxiety disorders in 204 countries and territories in 2020 due to the COVID-19 pandemic. *Lancet J.* 398 1700–1712. 10.1016/S0140-6736(21)02143-7 PMC8500697 34634250

[B21] Sønderskov K. M. Dinesen P. T. Santini Z. I. Østergaard S. D. (2020). The depressive state of Denmark during the COVID-19 pandemic. *Acta Neuropsychiatr.* 32 226–228. 10.1017/neu.2020.15 32319879 PMC7176490

[B22] Stathopoulou G. Powers M. B. Berry A. C. Smits J. A. J. Otto M. W. (2006). Exercise interventions for mental health: a quantitative and qualitative review. *Clin. Psychol. Sci. Pract.* 13 179–193.

